# Angiosarcoma of the Right Atrium Presenting as Syncope and Hemorrhagic Pericardial Tamponade

**DOI:** 10.1155/2012/829213

**Published:** 2012-11-05

**Authors:** V. G. Sams, A. Tsapenko, J. N. Kravitz, T. E. Gaines

**Affiliations:** ^1^Department of Surgery, University of Tennessee Medical Center, 1924 Alcoa Highway, Box U11, Knoxville, TN 37920, USA; ^2^Department of Pulmonary Medicine, University of Tennessee Medical Center, 1924 Alcoa Highway, Knoxville, TN 37920, USA

## Abstract

Angiosarcoma of the heart is a rare malignancy that can present in many ways. It is an important diagnosis to consider in patients presenting with otherwise unexplained tamponade-type symptoms. Here we present a case of a young male who presented with hemorrhagic tamponade and underwent resection of a large angiosarcoma of the right atrium. In this case, we describe the rare presentation of angiosarcoma with its diagnostic approaches, hospital course, clinical management, and discussion.

## 1. Introduction

Primary cardiac angiosarcoma is rare and aggressive with metastatic spread approaching ninety percent at the time of diagnosis [1]. It is the most common malignant tumor of the heart and is characterized by rapid growth, local invasion, and distant metastasis [2]. Multiple case reports have been published in the literature with presentations including pericarditis and pleuritis [3], chest pain and shortness of breath [4], and cough and hemoptysis [2]. Prognosis of patients with these primary tumors is very poor [5] with a mean survival of six months and poor chemotherapy response [6].

## 2. Case Report

This is a 27-year-old white previously healthy male with no past medical history and no risk factors for cardiovascular conditions who presented to the medicine critical care unit via interfacility transfer after an episode of chest pain with syncope while exercising and was hypotensive. He stated he had been experiencing some chest discomfort for several days with exercise intolerance and cold sweats.

### 2.1. Findings

Upon arrival, the patient was alert and in no acute distress with a heart rate of 112 and irregular and blood pressure of 112/77. His oxygen saturation was 94% on two liters of oxygen via nasal cannula. He had an obvious palpable pulsus paradoxus. All of his laboratory values were within normal limits with the exception of a mildly elevated creatinine.

### 2.2. Diagnosis and Management

**Figure 1:**
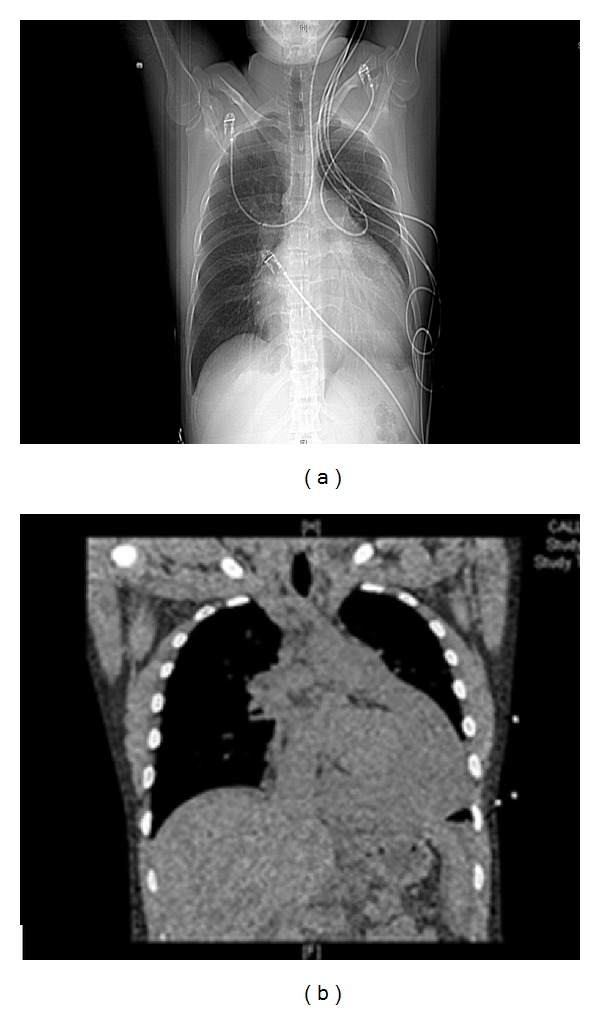
Radiographic imaging; chest X-ray (a) and CT scan (b).

Upon initial presentation, the patient received a therapeutic dose of enoxaparin for a presumptive diagnosis of pulmonary embolism. Following the drug's administration, further diagnostic workup included a chest X-ray which demonstrated cardiomegaly (Figure 1(a)), an echocardiogram which demonstrated a pericardial effusion with tamponade and a computed tomography scan also demonstrating the pericardial effusion (Figure 1(b)).

**Figure 2:**
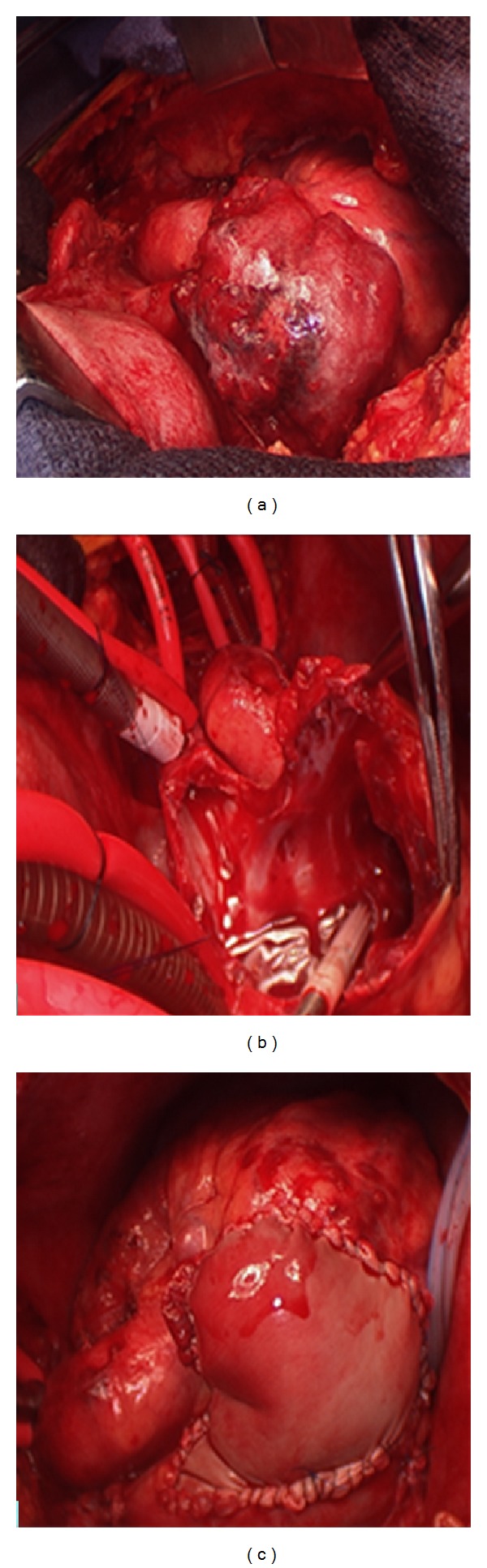
Intraoperative photos of identification (a), excision (b), and reconstruction (c) of the right atrial angiosarcoma.

Cardiothoracic surgery was consulted for pericardial drainage and possible biopsy. He was scheduled for a pericardial window the next morning since he had been anticoagulated on arrival and was hemodynamically stable after IV fluid administration. Intraoperatively, the patient was found to have a bloody pericardial effusion. The pericardial window did not allow adequate exposure to determine the source of the persistent bleeding. At this point we proceeded with a median sternotomy. Exposure of the heart revealed a large right atrial lobulated, bleeding mass. The pulmonary artery and aorta also had plaque-like lesions. Intraoperative frozen pathologic analysis suggested some type of high-grade angiosarcoma. We removed these lesions as well as performed an extensive node dissection to include pretracheal and right paratracheal lymph nodes. A decision was made to excise all gross disease, which involved the entire lateral wall of the right atrium, to best control the bleeding and prevent a recurrent effusion. The patient was placed on cardiopulmonary bypass. The mass was then resected to the right atrial and right ventricle junction next to the right coronary artery and including the sinoatrial node. We then used a bovine pericardial graft to reconstruct the atrium and placed two temporary right ventricular pacing wires (Figure 2).

The patient was extubated the following morning and managed with intravenous pain medication as well as pulmonary toilet. He did well and both mediastinal drains and right pleural drain were removed. He never required ventricular pacing and his final pathology was poorly differentiated angiosarcoma involving right atrial resection margin, virtually all of the lymph nodes, and the plaque-like lesions on the aorta and pulmonary artery. He was discharged to home with family with outpatient followup with medical oncology.

## 3. Discussion

Due to the rarity and aggressiveness of cardiac angiosarcoma, no curative treatment strategy has been developed and opportunity for meaningful intervention is slim. In this case, the goal of resection was to remove all gross disease in an attempt to eliminate the source of hemorrhage and tamponade. The other dilemma in evaluating these patients is the variability in cardiac angiosarcoma presentations. As seen in our patient, the presumption of pulmonary embolism with the administration of therapeutic enoxaparin could have worsened his bleeding and increased the degree of tamponade. Another differential diagnosis in a young, previously asymptomatic patient with cardiac tamponade is aortic dissection in which case enoxaparin would also be contraindicated. The disease is also difficult to diagnose with imaging when a complex bloody effusion is present. Surgery is the treatment of choice but often not curative. Patients often have metastatic disease at the time of surgery [7]. Aggressive and complete surgical resection offers the best palliation and prolongation of life [8]. Palliative resection in conjunction with chemotherapy and radiation can increase the length of survival and quality of life [9].

## 4. Conclusion

Primary cardiac angiosarcoma should be a consideration in the differential diagnosis of any patient with cardiomegaly associated with hemorrhagic pericardial effusion with tamponade.
